# Impacts of a Knowledge Mobilization Campaign for Carer-Inclusive Workplace Tools in Canada: Knowledge-to-Action Design Study

**DOI:** 10.2196/82473

**Published:** 2026-03-11

**Authors:** Brooke Chmiel, Allison Williams, Hinal Pithia

**Affiliations:** 1 School of Earth, Environment and Society, Faculty of Science, McMaster University, 1280 Main Street West, Hamilton, ON, L8S 4L8, Canada, 1 4162193312; 2 McMaster Continuing Education, McMaster University, Hamilton, ON, Canada

**Keywords:** carer-inclusive, caregiving, employment, workplace, knowledge mobilization, knowledge translation, awareness campaign, quantitative, creating caregiver-friendly workplaces

## Abstract

**Background:**

Coupled with an aging population and lower fertility rates, there is a growing number of carer-employees (CEs), those balancing unpaid care with paid employment. Over 5.2 million Canadians are CEs juggling this dual role, often incurring negative impacts to their mental and physical health as a result. Given that unpaid care makes up 75% of care provided in Canada, the economic importance of supporting CEs extends to sustaining health care systems. Supporting and accommodating CEs in the workplace has not only been proved to be beneficial to the well-being of CEs but also to the organization through increased productivity and lower turnover rates. Despite the clear advantages of implementing carer-inclusive workplace practices (CIWPs) in the workplace, many organizations across Canada remain largely unsupportive of CE accommodations. The present study evaluated the impact of a knowledge mobilization (KMb) campaign.

**Objective:**

The primary objective of the 2-phased campaign was to raise awareness of CIWPs in Canada and increase the uptake of various tools designed to support the implementation of CIWPs. Phase 1 published 4 articles in leading national industry magazines geared toward the target audiences; Phase 2 entailed a webinar series based around Phase 1.

**Methods:**

This study uses a quantitative methodology using data collected primarily through the various magazine article publishing companies, as well as project partner McMaster Continuing Education. Engagement metrics and analytics associated with each KMb activity were collected through social media platforms and website analytics. Tracking engagement metrics, such as views, unique views, social media impressions, social media clicks, registrations, and attendees, was used to evaluate the impact of the campaign.

**Results:**

The collected engagement metrics and analytics were analyzed to evaluate the campaign activities’ impact on increasing the engagement with and uptake of specific tools. Phase 1 activities brought in a total of 36,308 views (mean 9077, SD 17,336), 2469 unique views (mean 617, SD 548), 55,445 social media impressions (mean 13,861, SD 19,424), and 432 social media clicks (mean 108, SD 101) across all 4 articles. The most successful activity was Article 3, pitched toward the small- to medium-sized business audience. Phase 2 was successful in engaging the target audiences with the campaign materials to further promote and disseminate the tools. Webinar attendance rates varied across sessions (22.1%‐30.6%) with overlapping 95% CIs. Noticeable increases in engagement with the CIWP tools are observed during the months when Articles 3 and 4 were published.

**Conclusions:**

Results of the campaign suggest that published magazine articles targeted to the respective audiences are the most effective method of knowledge mobilization for this work, recognizing that paid activities had greater reach and better resources for dissemination. Future research in this area should focus on engaging with employers and professional stakeholders more directly.

## Introduction

### Background

The use of knowledge mobilization strategies for the dissemination of empirical research is becoming increasingly more prominent and crucial to many aspects of policy reform and development. While awareness of caregivers of adult dependents in Canada and their impact on society continues to emerge as a topic of concern economically, with a lens on the sustainability of health care systems in Canada, knowledge of how to support and accommodate these individuals for better health and well-being of both caregivers and care recipients remains to be seen more broadly.

### Carer-Employee Demographic

Many caregivers in Canada are balancing their unpaid care for an adult dependent with paid employment obligations; these folks are known as carer-employees (CEs). According to the 2018 Statistics Canada General Social Survey (GSS), approximately 5.2 million Canadians are CEs [1]. Coupled with increasing life expectancy rates and lower fertility rates, Canada’s population is aging rapidly, with the older adult (≥65 years) population in Canada expected to represent approximately 23% of the total population by 2030 [2-4]. With an increase in the numbers of those requiring care comes an increase in those needing to provide care; at the time of writing this, unpaid care makes up 75% of the care provided in Canada [5]. In addition to the majority of CEs working full-time employment positions [1], many provide around 5 hours of active care per day [6]. With this dual role come consequences to the overall health and well-being of both the CE and the care recipient. In particular, the intensity of balancing unpaid care responsibilities with paid employment can result in significant burnout, stress, fatigue, depression, and anxiety [7-12], which can lead to cardiovascular issues for the CE [13]. Analyzing the 2020 Carer Well-being Index, a study published in 2023 found that workplaces offering support and accommodation to their CEs during the COVID-19 pandemic resulted in better reported health and well-being by the CE. Specifically, from a 12-country comparison including the G7, Canada reported the least amount of support or accommodation from employers, and consequently, 71% of Canadian CEs reported worsening mental health and 52% reported negative impacts to their physical health [12]. Countries reporting higher levels of employer support for CEs also reported lower levels of negative impacts to mental and physical health [12]. As a result of juggling the dual role with little employer support, many CEs report feeling tired (80%), anxious (79%), and overwhelmed (73%) [12].

### Business Case

Despite the clear connections between supporting CEs in the workplace and reported positive impacts to overall well-being, many workplaces in Canada remain largely unsupportive of CEs in the workplace. This, in part, can be attributed to a lack of awareness of the benefits to the employer, or the “Business Case” for supporting these employees. Benefits to the employer surround return on investment (ROI) related to productivity, retention, reduced turnover, reduced leaves, and reduced absenteeism [9,14,15]. A report by the American Association of Retired Persons (AARP) found that for every dollar invested in flextime, organizations can expect an ROI ranging from US $1.70 to US $4.34, while investments in telecommuting yield returns between US $2.46 and US $4.45 [16]. According to the 2018 GSS, CEs are feeling the strain in balancing their paid employment and unpaid care role, with 46% reporting presenteeism at work, which refers to being physically present in the workplace but unable to concentrate or focus on the task at hand [1]. As well, 51% missed part of full days of work due to caregiving responsibilities, with 1 in 7 CEs reducing their paid work hours to accommodate the unpaid care role; consequently, 6% (approximately 214,000 Canadians) left or intended to leave the workforce due to caregiving responsibilities [1]. Carer-inclusive workplace practices (CIWPs) are highly variable and include the many different types of supports and accommodations that organizations can offer to better respond to the needs of CEs in the workplace [8,17,18]. CIWPs are carer-inclusive policies, programs, and/or practices that an organization implements to better support CEs in the workplace in the interest of improving their overall health and well-being, in addition to improving their productivity and engagement within the workplace [9,12,14,19]. Intervention studies have shown the effectiveness of CIWPs in not only improving the well-being and satisfaction of CEs in the workplace but also providing a tangible ROI to the organization in terms of reduced absenteeism and turnover rates with improved retention of highly skilled and experienced employees [15,16,20]. Despite these tangible benefits of CIWPs to both the employer and the employee, many organizations across different sectors are resistant to such implementation due to existing workplace culture [9,11,17,19,21,22]. Workplace culture is often cited as the most important factor in effectively realizing the positive change in a workplace environment for CEs [10,17-19,21,23-25].

### Gender Inequity

Globally, caregiving is often regarded as a women’s role and responsibility [26]. Women’s labor force participation is disproportionately impacted by unpaid care roles shaped by sociocultural norms [26,27]. While women have increasingly become a larger part of the workforce as many women enter the workforce in recent decades, the expectations around maintaining unpaid caring roles have remained the same [26,27]. This places significant strain on women’s ability to remain active participants in the workforce, often having to resort to reducing their paid work hours, declining promotions, and leaving the workforce altogether, resulting in women making up 60% of those who leave the workforce in Canada due to caregiving [1]. Although the proportion of CEs in Canada is approaching parity between men (48%) and women (52%), women still make up the bulk of unpaid care work, providing up to 3 additional hours per week on caregiving compared with men [1]. We know that the majority of CIWPs are offered in sectors dominated by women, primarily health care and education [18], while sectors dominated by men are often faced with more stigma and negative workplace culture around identifying as a caregiver and requesting support [22]. While the prevalence of CEs in Canada continues to grow, awareness of the negative impacts on their mental and physical health resulting from inadequate workplace supports remains largely invisible at the broader economic level [12]. Recognizing that unpaid care makes up 75% of care provided in Canada, with an estimated value of $24-$31 million (US $ exchange rate at time of study for all CAD amounts listed throughout this paper was US $ 1=1.3978 CAD) [5], supporting those balancing their unpaid care with their paid employment is economically critical not only for the sustainability of health care systems in Canada but also for continued labor force participation for women [28].

### Objective

Given the ongoing need to raise awareness about CEs in Canada and the tools and resources available to support them, this study evaluated a knowledge mobilization campaign geared toward increasing employer uptake of CIWP tools. The objective of this paper was to determine the most effective knowledge mobilization strategy for increasing uptake of the Creating Caregiver-Friendly Workplaces (CCFWP) online course, offered through McMaster Continuing Education (MCE) [29] and provides the opportunity to earn a microcredential, as well as the CSA (Canadian Standards Association) B701 Carer-Inclusive and Accommodating Organizations Standard (Standard) across workplace sectors in Canada and the associated B701HB-18 Helping Worker-Carers in Your Organization Handbook (Handbook) [30]. This study was also concerned with increasing visitors to the Gender, Health, and Work (GHW) program website, which houses a range of evidence-based tools and resources that were used to build the CSA B701 Standard and Handbook, as well as the CCFWP course [31]. Specifically, this paper aims to evaluate and compare engagement metrics across the various phases and activities of the knowledge mobilization campaign, addressing the research question, “What is the most effective knowledge mobilization strategy toward increasing the uptake of carer-inclusive initiatives across Canada?” Previous work in this area has emphasized the use of social media channels for the focus of knowledge mobilization work in this area [32]. Accordingly, this study explores both social media and other media channels to determine their impact on increasing engagement with and uptake of the noted tools.

## Methods

### Overview

**Figure 1.**
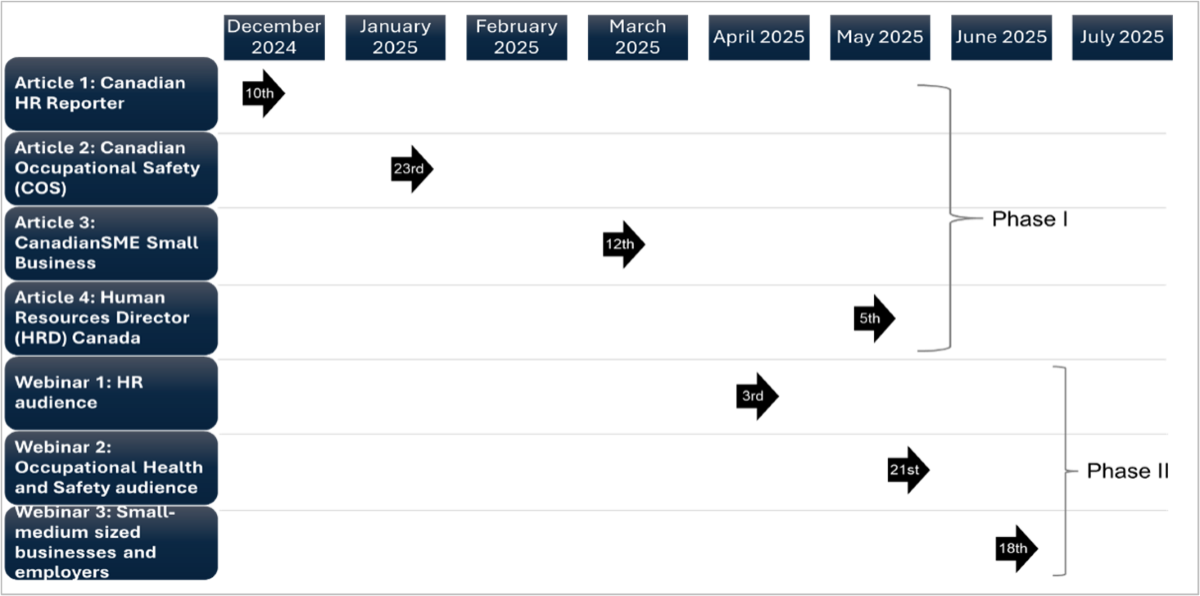
Timeline for Phases 1 and 2 of the campaign.

Through a Social Sciences and Humanities Research Council of Canada (SSHRC) Connection grant titled, “Disseminating tools for the creation of caregiver-friendly workplaces in Canada” (611-2024-0233), a knowledge mobilization campaign was conducted from December 10, 2024, to June 30, 2025. The campaign spanned 2 phases, Phase 1 being a series of 4 articles through various leading national professional magazines, and Phase 2 being a series of 3 webinars hosted by the project partner, MCE, virtually through Zoom (Zoom Communications). See Figure 1 below for a timeline of both campaign phases. Throughout both phases of the campaign, social media metrics were collected across each article and webinar through dissemination of the articles and webinars. A series of 4 infographics was created to boost the content of the first 3 articles, as well as the campaign products more broadly (ie, the CSA B701 Carer inclusive and accommodating organizations standard and the “Creating Caregiver-Friendly Workplaces” course).

### Analytical Framework and Campaign Evaluation Methods

Following a process model developed by Graham et al [33], the Knowledge to Action (KTA) approach is used to frame this knowledge mobilization (KMb) evaluation. To facilitate the uptake and use of knowledge, the KTA model provides a cyclical process for translating knowledge into action through mobilization of the knowledge [34,35]. The application of this cycle follows the process of identification of a problem (knowledge-practice gap) and the reciprocal knowledge to disseminate in order to implement a knowledge mobilization campaign (action) and finally assess knowledge use [33,34]. The action element of the cycle is the primary focus of this evaluation, bridging empirically supported knowledge about supporting CEs in the workplace into practice across target or key audiences (ie, Human Resources, Occupational Health and Safety leaders, and C-suite executives, and employers). This model shaped the selection of the target audiences for the campaign through the identification of a knowledge-practice gap in supporting CEs in workplaces across Canada and the stakeholders with the authority to implement the knowledge into practice within employment settings. This was also directly informed by results of a previous mixed methods evaluation surrounding the uptake of the CSA B701 Standard, with implications for future engagement with the identified audiences [24]. The 2-phased campaign design follows a multimodel KTA process that attempts to engage the audiences in various ways, persistently mobilizing the selected knowledge to the target audiences.

To effectively evaluate the outcomes of the KMb campaign with respect to increasing engagement with the selected knowledge, various statistical analysis methods are used to measure campaign results. The engagement metrics collected for the Phase 1 magazine publications, Phase 2 webinar series, and associated social media posts are analyzed using descriptive statistics (means and SDs) to summarize engagement. Attendance rates and 95% CIs for the webinar series were calculated using a normal approximation to the binomial distribution (ie, attendance was treated as a yes or no outcome). Excel (Microsoft Corp) formulas are used to calculate the descriptive statistics. The engagement metrics for the published magazine articles are provided by the media company “KeyMedia,” which manages 3 of the 4 selected industry magazines for the campaign (HR Reporter, HRD Canada, and Canadian Occupational Safety). The remaining metrics for the article directed at the employer audience were provided by said magazine, the CanadianSME. Details of the magazines with regard to reach and audience are outlined in the sections below.

#### Recruitment

Recruitment for the webinars was primarily conducted through the Customer Relationship Management (CRM) marketing software used by MCE (Microsoft); the 3 different audiences were strategically targeted through emails sent out by the MCE CRM database, which targeted those who had previously engaged in content or courses related to either human resources (HR) professional development or occupational health and safety, as well as small business owners and employers. The reach to the target audiences varied, shaped by MCE’s scope of access to the audience through previous content, courses, and webinar offerings. For the articles, total counts of views, unique views, social media impressions, and clicks were collected and were not disaggregated by channels. Each article was promoted through a month-long campaign across the magazines’ networks and social media channels.

#### Content Dissemination

Through KeyMedia, the first article was published in the Canadian HR Reporter magazine, a leading national magazine for HR professionals and managers to keep up to date on the latest trends in HR leadership and management, as well as how HR must respond to these societal changes. The HR Reporter magazine reaches approximately 30,000 HR professionals daily across the country; their website receives a monthly average of 200,000 page views and 124,000 users, as well as 15,000 email subscribers and 17,600 social media subscribers. Their audience is comprised primarily of female readers (79%), with 61% involved in product and/or service recommendation and 39% being end decision-makers for purchasing. A key focus of this magazine’s audience is employment law and workplace legislation, with 28% of their readership being managers of HR.

The second article was published in the Canadian Occupational Safety magazine, managed by the same media company, which reaches around 25,000 health and safety professionals daily. Their website receives a monthly average of 80,000 page views and 50,000 users, with 13,000 email subscribers and 30,000 social media subscribers. A large portion of their audience consists of health and safety managers and directors (41%), with 25% being health and safety coordinators or specialists. Most of their readership is based in Ontario, Canada (52%).

The third article was published in the CanadianSME Magazine, a member of Magazines Canada. The CanadianSME reaches around 30,000 readers per month across the country with 80,000 monthly impressions, 100,000 website visitors per month, and over 60,000 subscribers and followers on social media. The CanadianSME magazine connects 30 different business chambers across the country to provide the most up-to-date information to small business owners and employers. Most of their readership is also based in Ontario, Canada (52%), with 62% of readers being small business owners, founders, chief executive officers, and directors, and 27% being presidents and vice presidents. With regard to business type, around 40% of their readership is in retail business or manufacturing, both typically physically demanding occupations.

The fourth and final article was published in the Human Resources Director Canada (HRD Canada) magazine, a leading national magazine for stakeholders in HR and recruitment. According to their readership, the primary industries are education (16%), software (14%), manufacturing (13%), and health care (13%), with most readers (48%) representing HR directors, vice presidents, executive vice presidents, and senior vice presidents, while 28% of readers are heads of talent and recruitment. The fourth article was also managed by KeyMedia and collected the same metrics as Articles 1 and 2. Article 3 collected slightly different metrics than the KeyMedia articles, although highlighting similar engagement metrics of total reach, post engagement, and link clicks to either the article itself through social media or clicks within the article to the CCFWP course.

Registrations, attendees, and polling results were collected for the webinars. Webinars 1‐3 were framed around the content from articles 1‐3 and directed at these 3 audiences (HR professionals, occupational health and safety professionals, and small business owners and employers), respectively. Social media engagement across the articles and webinars was collected for both GHW and MCE social media accounts. To measure the success of these campaign strategies in increasing uptake of carer-inclusive initiatives across Canada, the downloaded data for the Standard and Handbook were collected from the CSA, as well as the student enrollments and microcredential earnings from the MCE course. The total number of students, microcredentials, and Standard and Handbook downloads were evaluated across the various campaign activities in terms of determining which method was most successful for boosting engagement with carer-inclusive initiatives.

### Ethics Considerations

For purposes of this study, the researchers were not required to obtain research ethics approval through the McMaster Ethics Research Board (MERB) as this study did not collect any personal or sensitive information from human participants. Metrics collected from the campaign reflect publicly accessible activities available to any individual across Canada. As these study activities, such as reading a magazine publication online or attending a webinar (both anonymously), were open to the public, the study did not directly engage with recruited human participants. Metrics that were provided by KeyMedia and CanadianSME resulted from the published magazine articles and present information on the total number of views and clicks for the article itself on the public web page, with no identifying information collected. As well, the webinars were publicly accessible events for any individual across Canada to register and attend, with only information on total attendees and click rates recorded (ie, no identifying information was asked for or collected).

## Results

Results of the knowledge mobilization campaign evaluation are outlined below, across the 2 phases.

### Phase 1

Table 1 below outlines the Phase 1 article metrics across the various magazines (managed and collected by KeyMedia and CanadianSME). Article 1 was published on December 10, 2024, and collected campaign metrics through to April 3, 2025. As part of the native article publishing package, KeyMedia runs a month-long paid campaign to boost the efforts of article dissemination to the key audience, although metrics collection continues following this date. Throughout this time, the article collected 524 views, 431 unique views, 3863 social media impressions, and 62 social media clicks. With regard to views, of the 431 unique users who viewed the article, 93 individuals revisited the article a second or more times. Of the 3863 social media users who viewed the post about the article in the HR Reporter magazine, 62 individuals clicked on the links associated with the article from the social media post, bringing the user to the article. Through project partner MCE, the article was also posted to MCE news on the MCE website. The article mirrored the version published on HR Reporter, with a link to that article provided at the end of the MCE post. The results were measured from December 13, 2024, to July 11, 2025, for a total of 204 views (includes those who may have revisited the article more than once).

**Table 1.** Article details and engagement metrics from Phase 1 of the knowledge mobilization campaign.

Source	Title	Link	Dates (data collection)	Views	Unique views	Social media impressions	Social media clicks
HR Reporter	Caring for carer-employees: an ethical imperative	[36]	December 10, 2025-April 3, 2025	524	431	3863	62
Canadian Occupational Safety	Championing caregiver-friendly workplaces as an occupational health and safety issue	[37]	January 24, 2025-March 10, 2025	271	236	5819	43
CanadianSME	How small businesses can use carer-inclusive initiatives to help their business thrive	[38]	March 12, 2025-April 3, 2025	35,080	1430	42,937	258
HRD Canada	“People will punch out one way or another”: the risk of ignoring carer-employees	[39]	May 5, 2025-July 4, 2025	433	372	2826	69

Article 2, directed to the occupational health and safety audience, was published on January 24, 2025, and measured metrics up until March 10, 2025. The KeyMedia campaign for the article collected 271 total views (including those who chose to revisit the article), 236 unique views, 5819 social media impressions (including unique users and those who chose to revisit the social media post), and 43 social media clicks. Of the 236 unique views of the article, 35 users chose to revisit the article, potentially sharing it with their networks. As noted in the methodology section, Article 3 collected slightly different metrics than Articles 1, 2, and 4. However, it paints a similar picture across Facebook (Meta Platforms, Inc), Instagram (Meta Platforms, Inc), X, formerly known as Twitter (X Corp), and LinkedIn (LinkedIn Corporation), where the article had a reach of 42,937, with a total of 258 social media clicks to the article web page. With regard to the article itself, across the e-magazine editorial published in March 2025 and the article web page on the CanadianSME Small Business magazine website, a reach of 35,080 was achieved, with a total of 1430 unique users viewing the article, 170 of whom clicked links within the article, driving readers to the CCFWP course web page for more information and registration for the course.

### LinkedIn

#### Overview

The article had a reach of 42,937, with a total of 258 social media clicks to the article web page. With regard to the article itself, across the e-magazine editorial published in March 2025 and the article web page on the CanadianSME Small Business magazine website, a reach of 35,080 was achieved, with a total of 1430 unique users viewing the article, 170 of whom clicked links within the article, driving readers to the CCFWP course web page for more information and registration for the course.

Finally, Article 4, published in HRD Canada magazine, targeted the HR audience and was published on May 5, 2025, and collected engagement metrics from the date of publishing through to July 4, 2025. The article’s campaign resulted in a total of 433 views, 372 unique views, 2826 social media impressions, and 69 social media clicks. Of the 372 unique views, 61 users chose to revisit the article.

In Table 2, the means and SDs were calculated across the totals of views, unique views, social media impressions, and social media clicks for all 4 articles. The average number of views for Phase 1 was 9077, although this is significantly skewed by the total views for article 3. The average number of views across all articles (with Article 3 [35,080 views] removed) is 409, compared with 9077 if Article 3 is included in the average. The mean unique views across all articles is 617, with an average of 13,861 social media impressions and 108 average social media clicks. The SD emphasizes the significant differences between views (SD 17,336), unique views (SD 548), social media impressions (SD 19,424), and social media clicks (SD 101) between articles 1, 2, and 4, and article 3.

**Table 2.** Descriptive statistics for total views, unique views, social media impressions, and social media clicks across the 4 articles.

Article number	Title	Dates (data collection)	Views	Unique views	Social media impressions	Social media clicks
1	Caring for carer-employees: an ethical imperative	December 10, 2025-April 3, 2025	524	431	3863	62
2	Championing caregiver-friendly workplaces as an occupational health and safety issue	January 24, 2025-March 10, 2025	271	236	5819	43
3	How small businesses can use carer-inclusive initiatives to help their business thrive	March 12, 2025-April 3, 2025	35,080	1430	42,937	258
4	‘People will punch out one way or another’: The risk of ignoring carer-employees	May 5, 2025-July 4, 2025	433	372	2826	69
Total	—^a^	—	36,308	2469	55,445	432
Average, mean (SD)	—	—	9077 (17,336)	617 (548)	13,861 (19,424)	108 (101)

a Not applicable.

**Figure 2.**
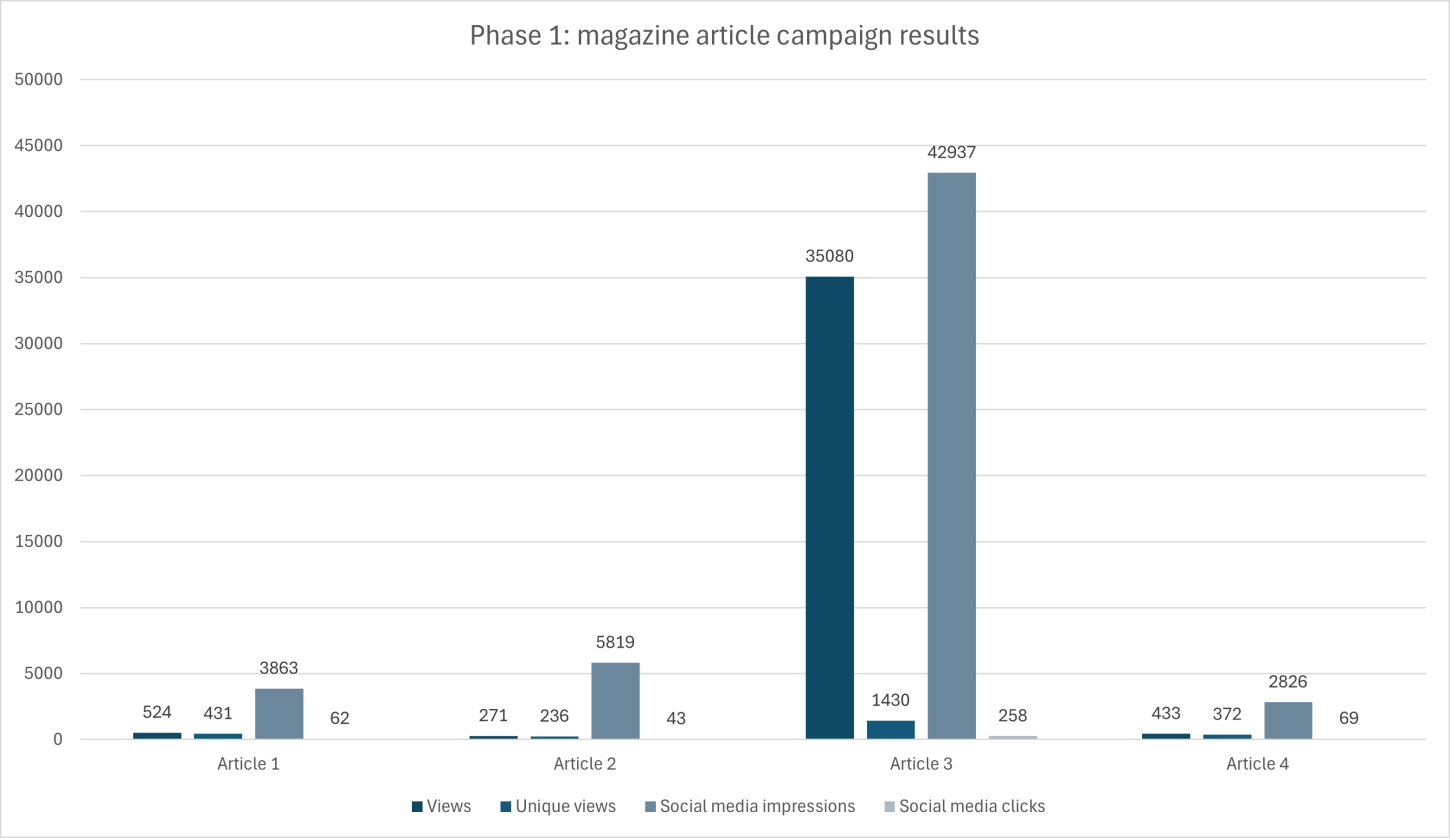
Engagement metrics across Articles 1‐4.

Figure 2 below provides a striking visualization of the overwhelming success of Article 3 for total views and social media impressions in comparison to the other articles.

In addition to collecting engagement metrics via the magazines directly, campaign partner MCE also collected engagement metrics for the articles through the posts to their website. The first article was published to the MCE website on December 10, 2025 collecting engagement metrics from December 13, 2024, to July 11, 2025, for a total view count of 204 (including unique views and overall views). Article 2 was published to the MCE website on January 31, 2025, collecting engagement metrics from January 31, 2025, to July 11, 2025, for a total of 114 views, while Article 3 measured results from May 7, 2025, to July 11, 2025, for a total of 136 views, and Article 4 a total of 81 views. Article 4 only measured views for the period of May 15, 2025-July 11, 2025 having been published to the website on May 15, 2025. Organic social metrics were collected for Articles 2‐4 (timing and capacity for MCE resulted in no organic social metrics collected for Article 1), as outlined in Table 3, which reports the total impressions and views, reach, and engagements across articles 2‐4. The averages and SDs for social media engagement across each article (2-4) are also included in Table 3 below. Overall, the most impressions and views collected across each platform for all articles were 495 Instagram impressions for Article 4. Article 3 had the greatest reach recorded across each of the articles and all social platforms, with 2935 being the total reach for X (formerly Twitter). Article 4, published in HRD Canada magazine, resulted in the greatest number of engagements recorded, recording a total of 13 engagements on Instagram. Across all social media platforms, X and Threads (Meta Platforms, Inc) had the highest percentage of engagement at 7% and 9%, respectively. Across all articles that recorded organic socials through the MCE website (Articles 2‐4), Instagram resulted in the highest number of impressions (n=1134) and engagements (n=35), while X resulted in the highest reach (n=7563). It should be noted that the platforms LinkedIn and Threads do not record the total reach, and therefore this number is excluded from Table 3 below.

**Table 3.** McMaster Continuing Education (MCE) social media engagement metrics, means, and SDs for Articles 2‐4.

Social platform	Article 2			Article 3			Article 4		
	Views	Reach	Engagements	Views	Reach	Engagements	Views	Reach	Engagements
Facebook, n	334	328	9	369	359	5	193	189	3
Instagram, n	228	188	12	411	296	10	495	343	13
LinkedIn, n	127	—	9	164	—	3	198	—	12
X, n	25	2314	1	64	2935	6	71	2314	4
Threads, n	9	—	1	5	—	1	8	—	0
Total, n	723	2830	32	1013	3590	25	965	2846	32
Mean (SD)	145 (138)	943 (1189)	6 (5)	203 (181)	1197 (1506)	5 (3)	193 (187)	949 (1185)	6 (6)

Table 4 provides the means and SDs for the social platforms used in terms of views and impressions, reach, and engagement across all of the articles (2-4), clearly indicating Instagram had on average the greatest number of views or impressions (average views: mean 378, SD 137), while X had the highest reach (average reach: mean 2521, SD 359). Across the board, engagement was fairly low (as noted above) in comparison to views and impressions and reach. Instagram had the highest average engagement (average engagements: mean 2, SD 2).

**Table 4.** McMaster Continuing Education (MCE) social media engagement means and SDs across the social media platforms.

Social platform	Average views, mean (SD)		Average reach, mean (SD)		Average engagements, mean (SD)	
Facebook	299 (93)		292 (91)		6 (3)	
Instagram	378 (137)		276 (79)		12 (2)	
LinkedIn	163 (36)		—^a^		8 (5)	
X	53 (25)		2521 (359)		4 (3)	
Threads	7 (2)		—		1 (1)	

a Not available.

#### Phase 2

Phase 2 of the campaign collected data related to the webinar series’ impact on increasing engagement with the CSA Standard, Handbook, and CCFWP course in a variety of ways. Data collection for this phase of the study was led by SSHRC Connection partner MCE. Each webinar was hosted through MCE’s Zoom platform, recorded, and uploaded to MCE’s YouTube (Google LLC) channel, as well as the GHW website. Through paid promotions and targeted webinar recruitment, MCE used various methods to bring in webinar registrants, corresponding with the target audience for each webinar. Table 5 below outlines the webinar series, including the title, link to the webinar recording, date of the event, total registrations, and total attendees.

**Table 5.** Phase 2 details of the webinar series.

Source	Title	Recording link	Dates	Registrations	Attendees
McMaster Continuing Education	Caring for carer-employees: an ethical imperative	[40]	April 3, 2025	272	60
McMaster Continuing Education	Championing caregiver-friendly workplaces as an occupational health and safety issue	[41]	May 21, 2025	170	52
McMaster Continuing Education	Carer inclusive employment policies: a strategy for small and medium-sized business success	[42]	June 18, 2025	120	32
Average, mean (SD)	—^a^	—	—	187 (77)	48 (14)

a Not applicable.

Table 6 provides the attendance rates and 95% CIs calculated separately for each webinar using a normal approximation to the binomial distribution (ie, “yes” or “no” for webinar attendance). Attendance rates ranged from 22.1% to 30.6%, with overlapping 95% CIs, indicating similar proportions of registrants attended each webinar despite the audience differences.

**Table 6.** Phase 2 webinar attendance rates and 95% CIs.

Webinar	Registrations, n	Attendees, n	Attendance rate, n	95% CI	
Webinar 1	272	60	0.2206	0.1713-0.2699	
Webinar 2	170	52	0.3059	0.2366-0.3751	
Webinar 3	120	32	0.2667	0.1875-0.3458	

**Figure 3.**
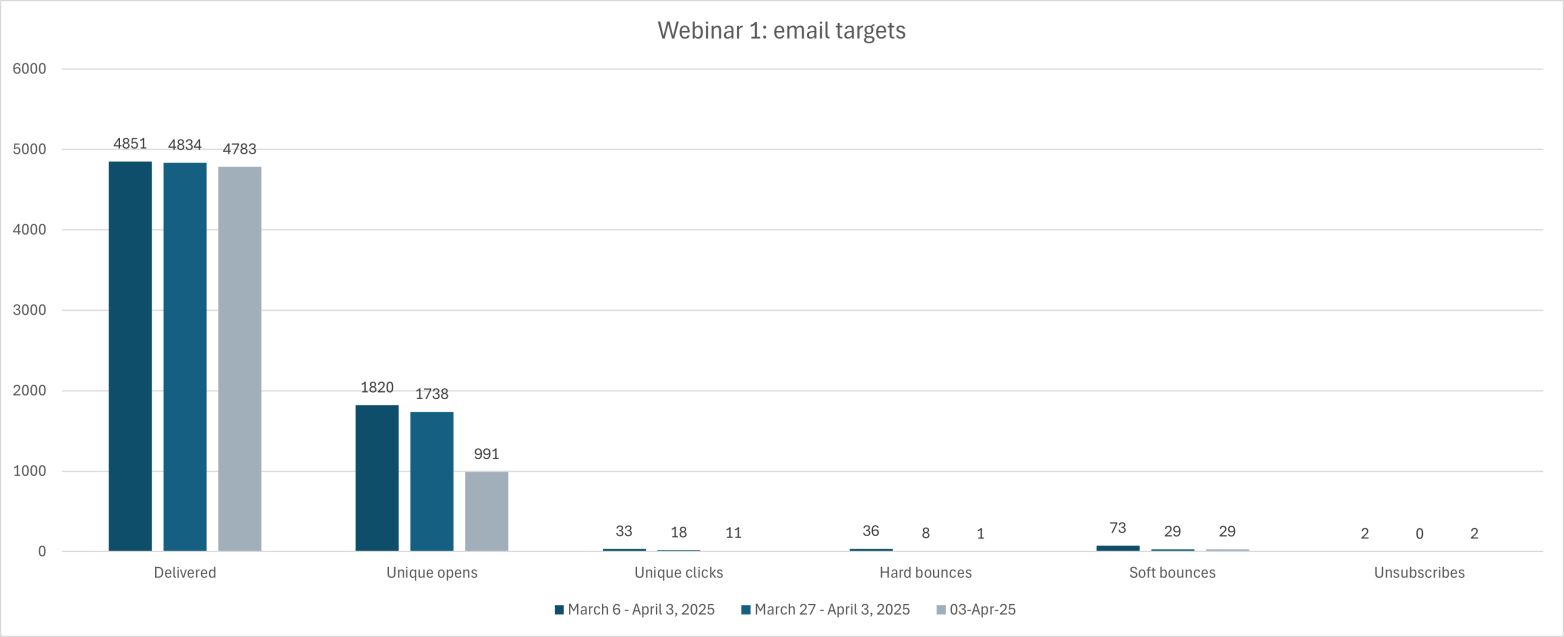
Email targeting metrics from Webinar 1, collected through McMaster Continuing Education (MCE).

Webinar 1 was held on April 3, 2025, and was built around Article 1 from Phase 1, directed toward the HR audience both at the managerial and C-suite levels. There were a total of 272 registrants on the day of the webinar, of which 60 attended the webinar. Primarily, email targets were used to connect with those who had previously engaged in one of the other HR-related programs offered through MCE, whether that be a course, program, or webinar. A total of 6427 individuals were targeted through the email CRM system, 59 of whom were MCE staff for a testing segment. MCE students actively enrolled and/or graduates of the Human Resources Management and Business Administration programs were targeted. Of the 6427 emails sent out, 4851 were delivered for Invite 1, with 1820 unique opens of the email (meaning 3031 people did not open the email) and 33 unique clicks (meaning 1787 people did not click any links within the opened email). For Invite 2, a total of 4834 were delivered, with 1738 unique opens and 18 clicks to register for the webinar. The third invite was sent out the morning of the webinar as a last call and reminder, with 4783 emails delivered, 991 unique opens, and 11 clicks. Figure 3 below provides a visual of the significant difference between the total amounts of those invitations delivered and the resulting clicks to register for the webinar.

**Figure 4.**
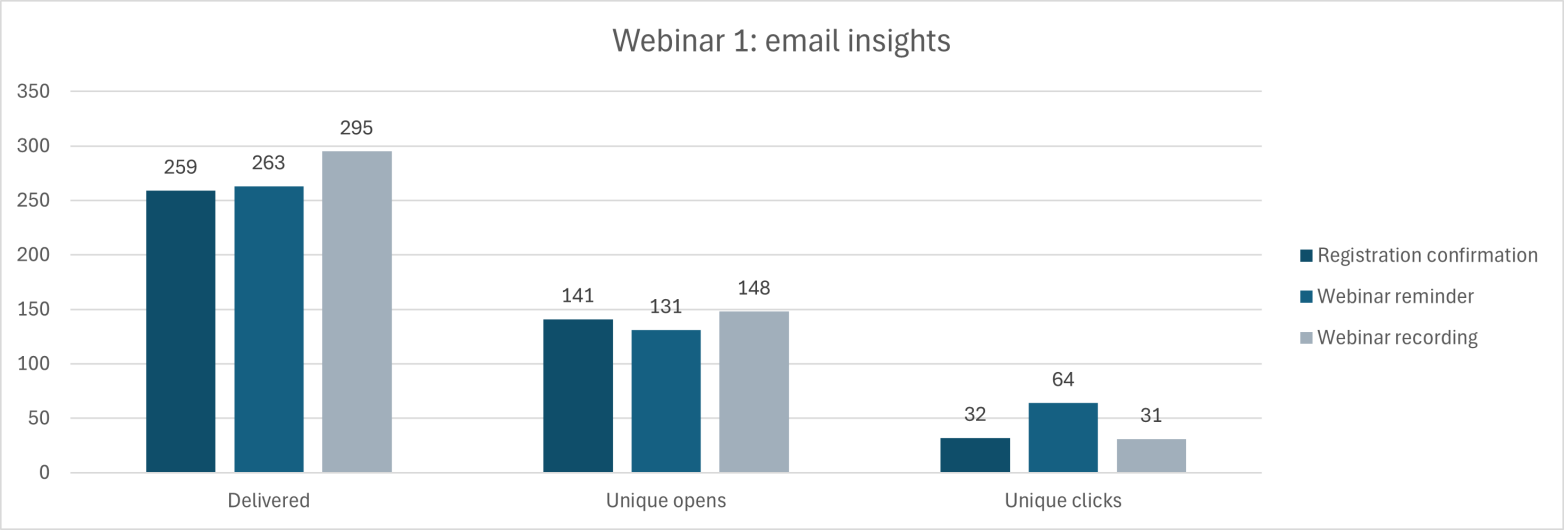
Metric insights from the email targets sent out by McMaster Continuing Education (MCE) to recruit for Webinar 1.

With regard to those who registered for the webinar, Figure 4 below visually outlines the total of emails delivered regarding the registration confirmation, a webinar reminder, and the webinar recording following the live webinar. About half of those who received each email opened the message, and a smaller portion of unique clicks within the opened email (the highest of which being those who clicked on the link to the webinar for the reminder email).

Paid social media advertisements were also deployed as part of the MCE marketing campaign for the webinar series through Meta Platforms, including Facebook and Instagram. The campaign featured a Facebook boosted event and a lead generation ad targeting users on both platforms. The paid social media advertisements for Webinar 1 measured impact from March 6, 2025, to April 3, 2025 (ie, “in market” timeline), with a budget of $150 CAD for the Facebook boosted event and $900 CAD for the lead generation ad. The Facebook boosted event resulted in 97 event responses, with a reach (unique users) of 9576 and 27,720 impressions (total times the ad was viewed, ie, unique users revisiting the ad more than once). The lead generation promotion resulted in 162 leads (ie, driving users to register for the webinar), with a reach of 27,008 and 74,488 impressions (see Table 7 below).

**Table 7.** Paid social media advertisements through McMaster Continuing Education (MCE) platforms (Facebook and Instagram).

Type	In-market timeline	Budget	Results	Reach	Impressions	Cost per result
Boosted Facebook event	March 6, 2025-April 3, 2025	CAD $150	97 Event Responses	9576	27,720	$1.55 Per event response
Lead generation Ad	March 6, 2025-April 3, 2025	CAD $900	162 Leads	27,008	74,488	$5.55 Per lead

**Figure 5.**
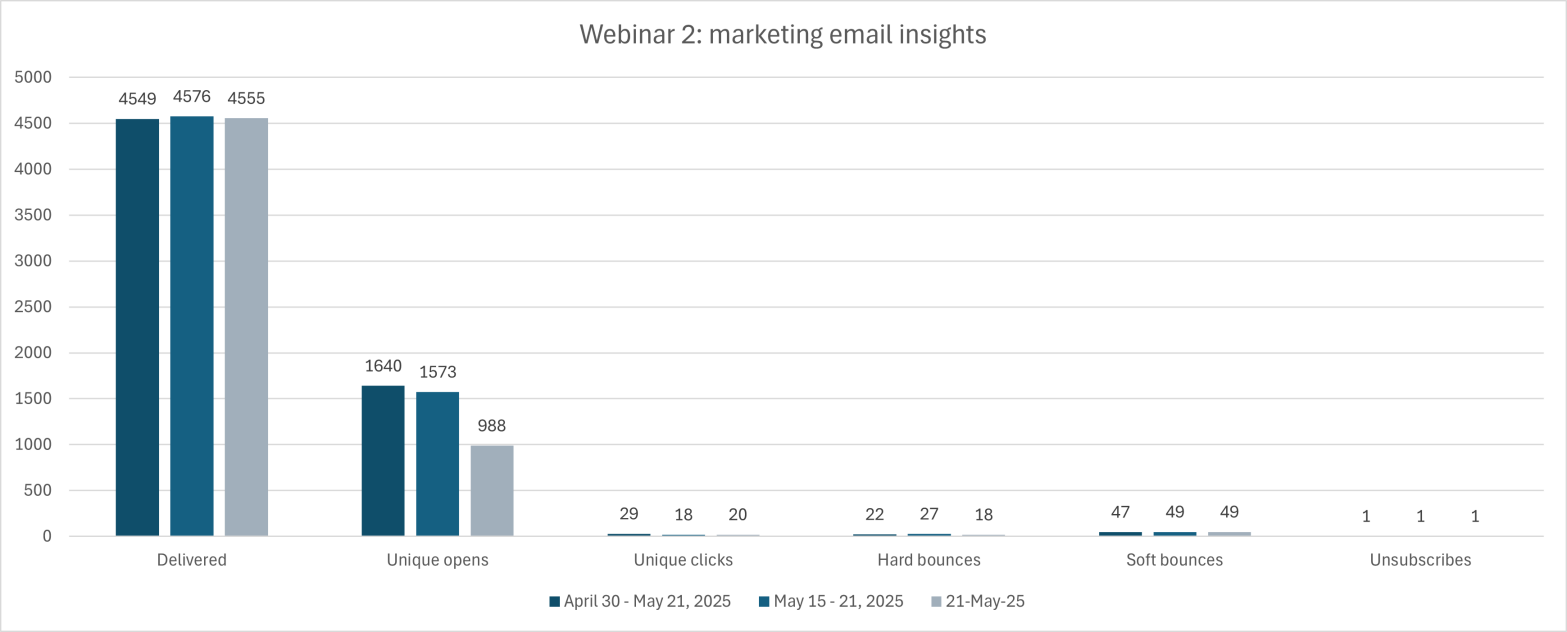
Marketing email insights of those contacted by McMaster Continuing Education (MCE) for Webinar 2 recruitment.

**Figure 6.**
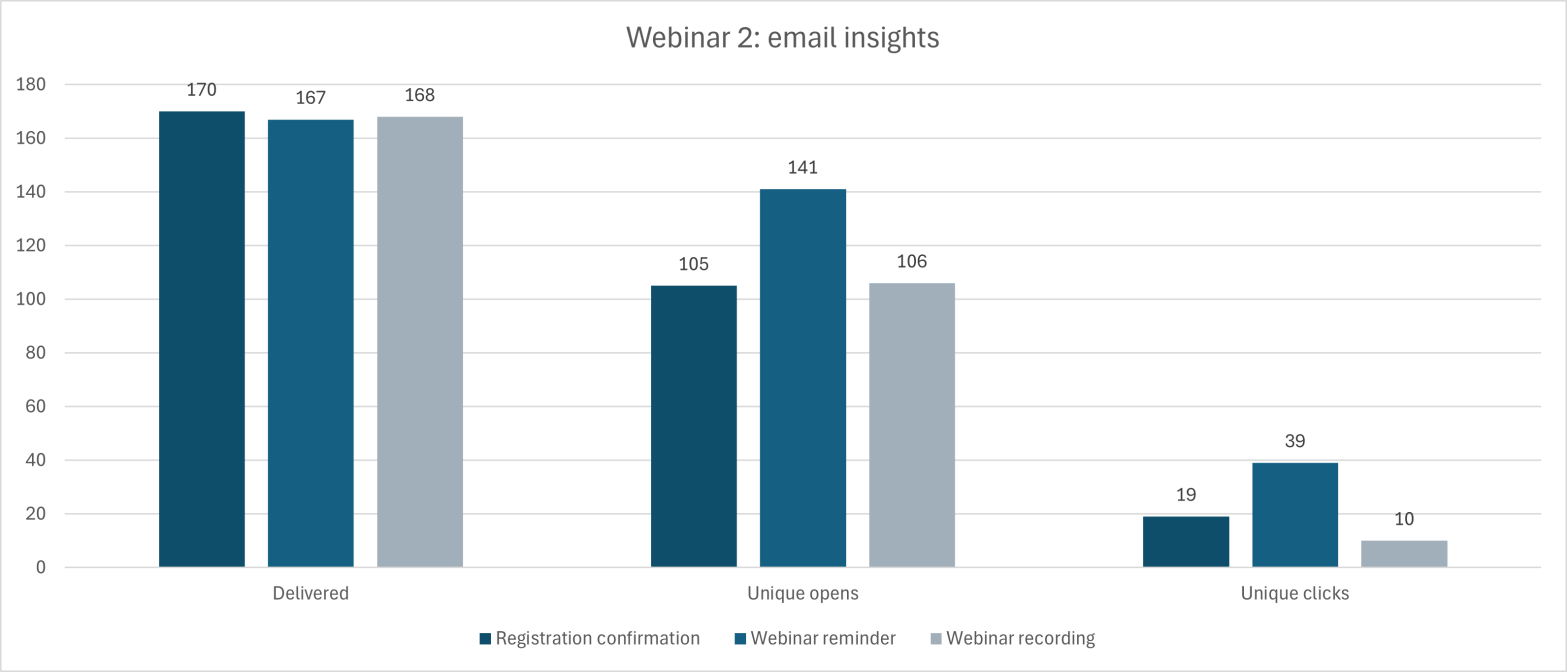
Metric insights from the email targets sent out by McMaster Continuing Education (MCE) to recruit for Webinar 2.

Webinar 2, held on May 21, 2025, brought in a total of 170 registrations and 52 attendees. MCE contacted a total of 6159 individuals through the CRM email marketing, 59 of those being MCE staff members for a testing segment. Geared toward the Occupational Health and Safety (OHS) audience, those active in or graduates of the Human Resources Management and Business Administration were again targeted, as there are no existing OHS programs through MCE. Three invites were sent as part of the targeted recruitment, the first capturing data from April 30, 2025, to May 21, 2025, for a total of 4549 emails delivered, 1640 unique opens of the email, and 29 unique clicks of the registration link to the webinar. Invite 2 collected data from May 15 to 21, 2025, with a total of 4576 emails delivered, 1573 unique opens, and 18 clicks of the registration link within the email. The third invite, sent out the day of the webinar, resulted in 4555 emails delivered, 988 unique opens, and 20 unique clicks (see Figure 5 below). The registration confirmation email measured delivery, unique opens, and unique clicks from April 23, 2025, to May 21, 2025, resulting in 170 delivered, 105 unique opens, and 19 unique clicks. The webinar reminder collected data on May 21, 2025 alone and resulted in 167 delivered, 141 unique opens, and 39 unique clicks. The email following the webinar regarding access to the webinar recording measured reach from May 23, 2025, to June 16, 2025 and resulted in 168 emails delivered, 106 unique opens, and 10 unique clicks. Figure 6 provides a visual of the decline in terms of ratio across delivered, opened, and clicked, with less than half of those who opened the email clicking on an embedded registration link.

A boosted Facebook event and a lead generation ad were deployed as a recruitment strategy, collecting data from April 24, 2025, to May 20, 2025. The Facebook boosted event was budgeted at CAD $100, resulting in 65 event responses, reaching 6846 and 16,852 impressions. The lead generation ad was budgeted at CAD $900 and resulted in 110 leads (people who gave their contact information to register), with a reach of 32,969 and 93,172 impressions. The cost per result (CPR), which refers to the average cost per specific action throughout the paid advertisement campaign, for the Facebook Boosted Event was valued at $1.54, and the Meta Lead Generation Ad had a CPR of $8.18 (see Table 8 below).

**Table 8.** Paid social media advertisements through McMaster Continuing Education (MCE) platforms (Facebook and Instagram).

Type	In-market timeline	Budget	Results	Reach	Impressions	Cost per result
Boosted Facebook event	April 24, 2025-May 20, 2025	CAD $100	65 Event responses	6846	16,852	$1.54 Per event response
Lead generation Ad	April 24, 2025-May 20, 2025	CAD $900	110 Leads	32,969	93,172	$8.18 Per lead

**Figure 7.**
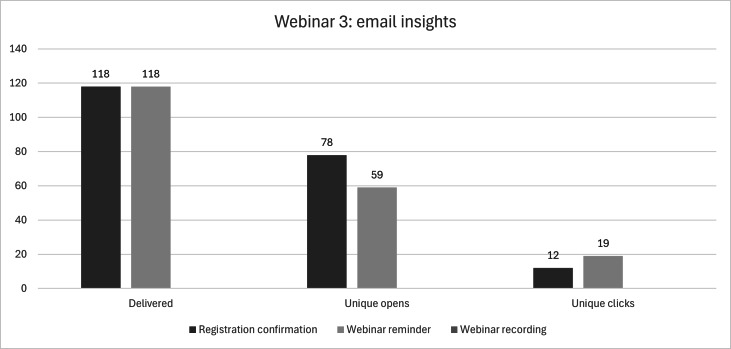
Metric insights from the recruitment emails sent out by McMaster Continuing Education (MCE) for Webinar 3.

Webinar 3 was pitched toward the small- to medium-sized business audience and was correlated to Article 3 Phase 1. Planning logistics impacting the timeline of the webinar led to a shorter period between recruitment for the webinar and the actual date of the webinar, specifically around speaker confirmation and session organization. As such, the third webinar, hosted on June 18, 2025, only collected data on one email invite to those within MCE’s CRM for the period of May 23, 2025, to June 18, 2025. The webinar had a total of 120 registrations and 32 attendees. The invite was sent out to 22,174 individuals, targeted at those active at the time of this writing or a previous graduate in any of MCE’s business programs, with 16,047 delivered, 5562 unique opens, and 75 unique clicks. For those who had registered, a registration confirmation email was sent, and responses were measured during the period of May 9, 2025, to June 18, 2025, for a total of 118 emails delivered, 78 unique opens, and 12 unique clicks to the webinar link. The webinar reminder email was sent out the day of the webinar for a total of 118 delivered, 59 unique opens, and 19 unique clicks to the webinar link (see Figure 7 below). The paid social media advertisements for webinar 3 mirrored that of webinars 1 and 2, measuring impact on Meta’s social media platforms from May 22, 2025, to June 17, 2025. With a budget of CAD $100, the boosted Facebook event resulted in 70 event responses, with a reach of 6045 and 14,524 impressions at a rate of $1.43 cost per event response (or CPR). The lead generation ad, with a budget of CAD $900, generated 64 leads, reaching 13,886 individuals with a total of 49,445 impressions. For the lead generation ad, the CPR is significantly higher at $14.06 per lead (or CPR; see Table 9 below).

**Table 9.** Paid social media advertisements through McMaster Continuing Education (MCE) platforms (Facebook and Instagram).

Type	In-market timeline	Budget	Results	Reach	Impressions	Cost per result
Boosted Facebook event	May 23, 2025-June 17, 2025	CAD $100	70 Event Responses	6045	14,524	$1.43 Per event response
Lead generation Ad	May 22, 2025-June 17, 2025	CAD $900	64 Leads	13,886	49,445	$14.06 Per lead

Webinars 2 and 3 collected polling data during the live session from the audience members in order to gauge the impact of the webinar toward increasing uptake of the various CIWP tools and resources (primarily the CCFWP course and CSA B701 Standard and Handbook). Webinar 1 missed the opportunity to collect polling data, as the polls were launched too late into the session, following the question-and-answer period, when audience members had already logged off. This informed the structure of the remaining 2 webinars, where the polls were launched prior to the start of the question-and-answer period to ensure responses for the polls were collected before attendees began signing off from the session. Table 10 below provides an overview of the questions launched throughout Webinars 2 and 3, with polling question 2 from Webinar 2 belonging to the guest speaker for that session and not related to data collection of the knowledge mobilization campaign.

**Table 10.** Polling report summary for Webinars 2 and 3.

Poll name		Questions, n	Responses, n
Webinar 2 (registrations: 170; attendees: 52)			
Poll 1 - What is your position/role		1	18
Poll 2 - If you have been awake for 21 hours		1	29
Poll 3 - How likely are you to enroll in CCFW^a^		1	28
Poll 4 - How likely are you to pursue policies		1	27
Webinar 3 (registrations: 120; attendees: 32)			
CCFW^a^ Web 3 - Poll 1 - workplace sector		1	11
CCFW Web 3 - Poll 2 - workplace size		1	11
CCFW Web 3 - Poll 3 - Likely to enroll		1	9
CCFW Web 3 - Poll 4 - Pursue policies at work		1	11

a CCFWP: Creating Caregiver-Friendly Workplaces.

From the table, we can see that Webinar 2 generated the most responses to the polling questions, likely related to the number of registrations and attendees, as Webinar 2 had 20 more attendees than Webinar 3. For Webinar 2, each attendee was asked what their position, role, and title at their organization are at the time of this writing, with responses ranging from Health and Safety Manager, Officer, and Specialist to Consultant. A total of 28 responses were recorded for polling question 3, “How likely are you to enroll in the Creating Caregiver-Friendly Workplaces course?” as part of Webinar 2, with a Likert scale ranging from 1 to ‐10 (1 being the least likely and 10 being most likely), with 71% of respondents reporting 7 or higher and an average of 7 (SD 3) across the 28 responses. When asked, “How likely are you to pursue carer-inclusive initiatives or policy at your place of work?” a total of 27 attendees completed the poll, with 88% reporting 7 or higher and an average of 8 (SD 2), indicating Webinar 2 was successful in increasing potential engagement not only with the CCFWP course but with CIWPs more broadly. Webinar 3 asked a similar set of polling questions, starting with the workplace sector of the attendee. Given that Webinar 3 was geared toward the small (1‐99 employees) to medium (100‐499 employees) business audience, the polling question was more concerned with workplace sector as opposed to role, collecting a total of 11 responses ranging from finance and insurance to health and social services. The second question asked attendees about workplace size, with 100% of respondents (11 total) reporting from a small business. When asked how likely they were to enroll in the CCFWP course, 67% of the 9 respondents reported 7 or higher, with an average of 6 (SD 3). When asked how likely they were to implement CIWPs into their workplace, 91% of the 11 respondents reported 7 or higher, with an average of 8 (SD 3), indicating strong likelihood. This suggests Webinar 3 was also successful in increasing engagement with the tools and resources being disseminated as part of the KMb campaign.

Each webinar was recorded and posted to MCE’s YouTube channel as part of the “Program Webinars” series. Within the week following the live sessions, each webinar recording was uploaded to the YouTube channel, collecting views from the period of posting to the date of checking the total views, July 24, 2025. The first webinar, geared toward the HR audience, was posted to the channel on April 7, 2025, and has a total of 67 views. The second webinar, geared toward the OHS audience, was posted May 23, 2025, and has a total of 39 views. The third webinar has a total of 33 views and was posted on June 25, 2025. Despite having been posted to the channel for the shortest period of time, Webinar 3 has fared similarly well to Webinar 2, which could suggest the small- to medium-sized business audience may be more receptive to the issues of CIWPs than the OHS audience. It should be noted that the first webinar has been uploaded to the channel for a longer period of time than the most recent webinars, potentially contributing to a difference in total views.

### Impact on Uptake

**Figure 8.**
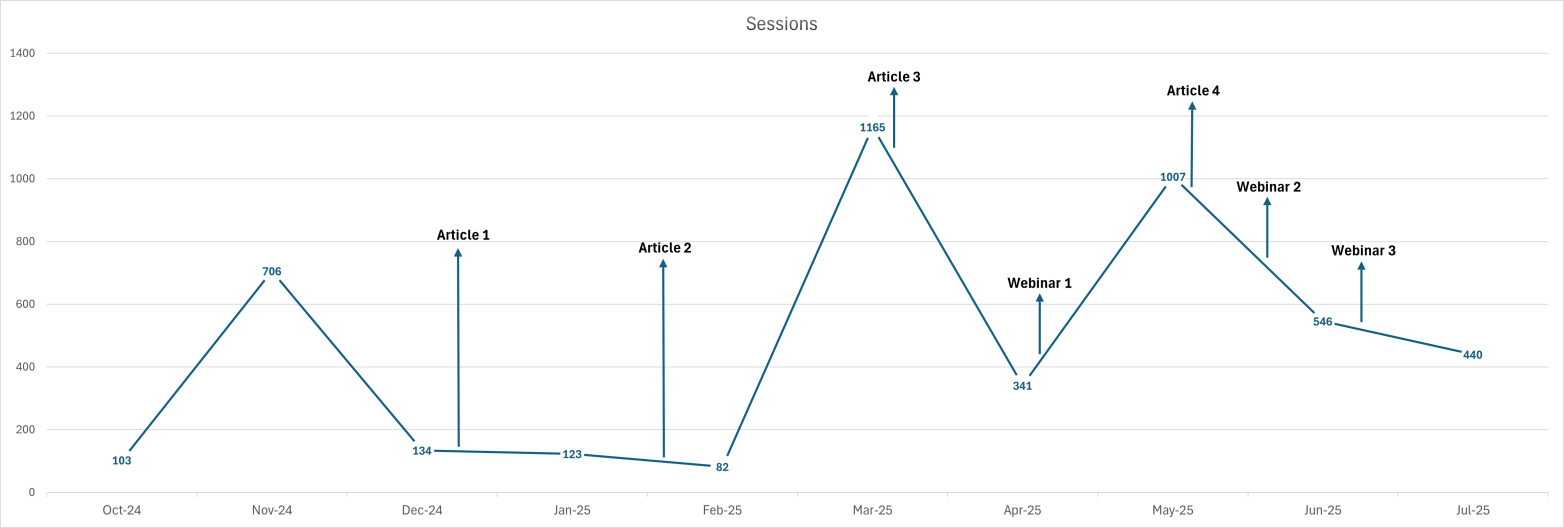
Sessions (visits) to the Creating Caregiver-Friendly Workplaces (CCFWP) web page throughout the months of the knowledge mobilization (KMb) campaign with Phases 1 and 2 activity indicators.

In relation to driving traffic to the CCFWP course web page for increasing registrations to the course with the end goal of increasing uptake of CIWPs, the articles published in Phase 1 had the most success in increasing engagement with the CCFWP course web page. The number of monthly “Sessions” to the CCFWP course web page refers to visitors to the site, where we specifically see spikes and increases in visits to the website around the time of article publishing. Although it is important to note that increases in visitors to the CCFWP course web page do not indicate direct engagement with the course itself but do, nonetheless, increase awareness of the course through an increase in traffic. As shown in Figure 8 below, the highest spike in visitors (n=1165) to the website throughout the month of March 2025 coincides with the publishing of Article 3 in the CanadianSME magazine on March 12, 2025. The second highest spike in visitors to the website throughout the month of May 2025 (1007) is observed around the same time Article 4 is published in the HRD Canada magazine, geared toward senior and executive HR representatives in C-Suite with stakeholder influence. This observation does not necessarily indicate the spike in visitors to the web page is a result of the article publications, although it is possible the articles influenced this increased spike in traffic to the web page. Interestingly, there is an observed spike in visitors to the website in the month of November 2024 (n=706), the month predating the first article being published in HR Reporter magazine. There are no campaign activities that occurred around this time to explain the spike in visitors to the CCFWP web page. Notably, there is a limited increase of traffic to the web page around the time of the second article being published in the Canadian Occupation Safety magazine.

**Figure 9.**
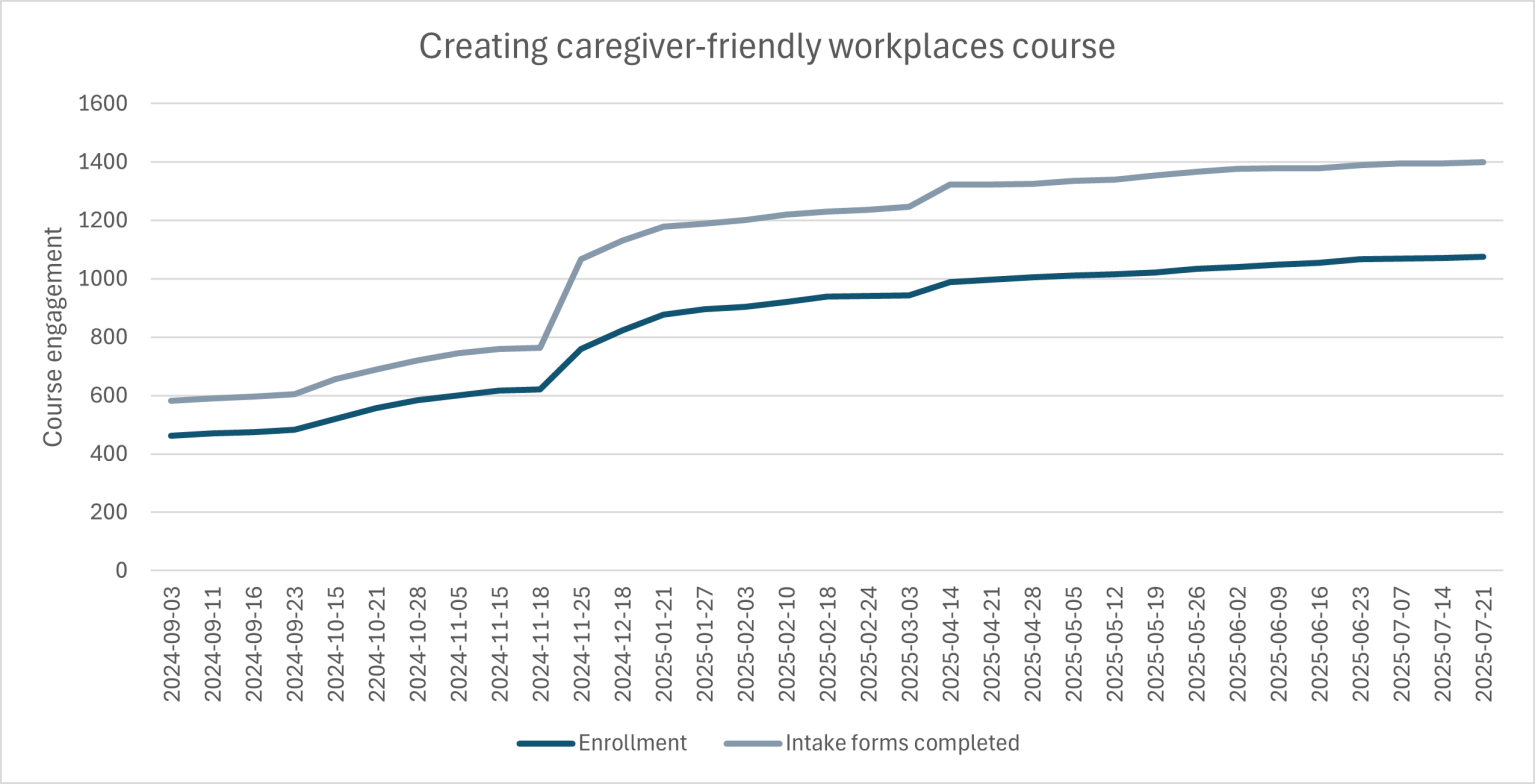
Registrations (intake forms completed) and student enrollments for the Creating Caregiver-Friendly Workplaces (CCFWP) course throughout the timeline of the knowledge mobilization (KMb) campaign with indicators of Phases 1 and 2 activities below the x-axis.

With regard to the CCFWP course and the impacts of the campaign on course enrollment and engagement, there are subtle yet noticeable spikes in activity following the publishing of Article 3 and the live session of Webinar 1. From that point onward, traffic to the CCFWP web page appears to sit above the norm prior to February, excluding the spike in visitors in November 2024. This could potentially be influenced by the campaign activities, although more in-depth data collection and analysis would be needed to determine the association. Article 3 published in the CanadianSME magazine appears to be the most successful KMb activity, with an increase in traffic to the CCFWP course web page occurring around the same time as article publication, as well as an observed increase in registrations and enrollment in the course. Second to Article 3, Article 4, published in HRD Canada*,* appears to potentially have contributed to the second-highest spike in sessions to the web page, with an observed gradual increase in registrations and enrollments in the CCFWP course throughout the month of May 2025. Recognizing the limited evidence, we cannot conclude that the KMb campaign directly impacted the noted increases in traffic to the CCFWPs and enrollments in the course. As shown in Figure 9 below, there is a significant spike in enrollment and intake forms completed throughout the month of November 2024, coinciding with the spike in visitors to the CCFWP web page as noted in Figure 8 above, with a lack of explanation for the activities as part of the KMb campaign.

Following the publishing of Articles 1 and 2, there is an observed spike in downloads to the Handbook specifically, while the Standard did not appear to increase in downloads following the start of Phase 1. One download of the Standard occurred in December 2024, the same month Article 1 was published, in comparison to zero downloads in the months predating Article 1. Although this does not suggest Article 1 influenced the Standard download. Following the publication date of Article 1, the Handbook received a total of 11 downloads throughout the month of December 2024, in comparison to just one download for both the months of October 2024 and November 2024. Article 2 was published January 23, 2025, coinciding with a total of 9 downloads of the Handbook throughout the month of January 2025. No articles were published in February 2025. There is a drop in downloads observed, with just 2 downloads for the month of February 2025. There was an increase in downloads of the Handbook throughout the month of March 2025, for a total of 5 downloads.

There is a spike in visitors to the GHW program website throughout March 2025. It should be noted that due to restrictions in access to website analytics for the GHW website, only the previous 6 months were available for metrics (February-July 2025). The total number of visitors to the website (n=1903) and collective views across the various sections of the website (n=31,099) are substantially higher than is normal for traffic to this website. The primary campaign activity that occurred during this time was the publishing of Article 3, although no direct association or correlation has been made.

## Discussion

### Principal Findings

This study evaluated the impact of a 2-phased knowledge mobilization campaign pitched toward increasing awareness of and engagement with selected tools and resources for curating carer-inclusive workplaces. Overall, the findings indicate that the activities of Phase 1, particularly targeted industry magazine publications, generated more engagement with the campaign tools than the webinar series of Phase 2. Engagement varied across the target audiences, with higher HR professional and employer audience engagement. These findings address the study objectives by identifying industry magazine publications as the most effective within this campaign. Increased visits to the CCFWP course web page, registrations and enrollments to the course, increased visits to the GHW course web page, and Standard and Handbook downloads were observed around key campaign activities directed at the HR and employer audiences. The use of multiple channels for KMb dissemination is a growing common practice in KMb initiatives across researchers and research groups [35,43-47]. Specifically, the use of targeted strategies that directly engage with select audiences.

Phase 1 consisted of the 4 paid magazine articles in leading Canadian industry magazines, pitched toward the target audiences (HR professionals, OHS professionals, and small- to medium-sized businesses). The most noticeable increase in both visitors to the course web page and increase in registrations and course enrollments, as well as increased visitors and traffic to the GWH website, is observed around the activities of Phase 1. This phase resulted in a total of 36,308 views, 2469 unique views, 55,445 social media impressions, and 432 social media clicks across all 4 articles between the period of December 10, 2024, and May 5, 2025. Of the 4 articles, Article 3 (small- to medium-sized business audience) had the highest ratio of views to unique views (35,080:1,430) and had the lowest proportion of unique views to views (4%). Article 2 (OHS audience) had the lowest ratio of views and unique views (271:236). However, 87% of the total views for Article 2 were unique views, in comparison to 86% and 82% for Articles 4 and 1, respectively. Article 3 was also superior to social media engagement across the 4 articles, again with the highest ratio of social media impressions to social media clicks (42,937:258). Surprisingly, the success of Article 3 surpassed that of Article 1, given that the HR Reporter has 100,000 more monthly visitors to their website than the CanadianSME magazine. This could potentially reflect that 98.1% of businesses in Canada are classified as small businesses, equating to over 1.07 million small businesses in Canada [48]. The significantly greater reach of Article 3 skews the results of Phase 1, with large SDs around the mean (average views: 9077, SD 17,336; average social media impressions: 13,861, SD 19,424).

Given the results of the overall KMb campaign analysis, the HR and small- to medium-sized business audiences are observed to have the most engagement with the campaign materials and activities. Activities directed toward these audiences resulted in the highest levels of engagement through the magazine Articles 3 (March 2025) and 4 (May 2025). Corresponding spikes in visits to the CCFWP course web page were observed, with 1165 and 1007 sessions recorded, respectively, as well as registrations and enrollments to the course. While there are visible spikes in website traffic around the publication dates of Articles 3 and 4, it is uncertain whether this is a direct result of the KMb campaign. However, it is possible the articles contributed to increased awareness of the CCFWP course given the increased traffic around these times. More sophisticated quantitative analysis is needed to determine more direct impacts of campaign activities on increased engagement with the selected tools and resources.

Despite Phase 2’s lower performance when compared to the impact of Phase 1, both in terms of views and engagement and observed traffic increases to the CCFWP website and GHW website, activities directed toward the HR and employer audiences received the highest engagement. Although Webinar 2 had greater registration and attendance than Webinar 3, the recording has garnered more engagement through the YouTube channel. With regard to the various social media channels, while X had the highest reach (7563 [82% of all reach across each article]) across all articles that collected organic socials (Articles 2‐4), Instagram was the most effective platform for collecting the most impressions (42%) and engagements (39%) across all social media channels and metrics for Articles 2‐4. This corroborates the findings of previous research in this area that found X to have the greatest reach for disseminating CE tools, while Instagram had the greatest depth of engagement (ie, longer engagement or interaction) [32]. While this study cannot establish the direct impact of the KMb campaign activities on the increase in activity to the CCFWP course web page and enrollments, GHW website, and Standard and Handbook downloads, future knowledge mobilization research in this area may benefit from an increased focus on the occupational health and safety audience as it appears to be more challenging to engage. It may be that alternative channels of outreach could be more successful in garnering engagement with campaign materials and activities with the OHS audience. Tools and resources may benefit from being better framed around psychological health and safety, and the implications of ignoring the challenges experienced by CEs in the workplace may be best disseminated through associated channels. Engaging with OHS stakeholders in future research can add value to existing research in the areas of KMb science specific to professional or technical audiences. Results of the campaign analysis show that the Handbook received more downloads when compared to the Standard throughout the duration of the campaign. This highlights results of a previous study that evaluated the uptake of the CSA B701 Standard and Handbook. The study found that the Handbook is often perceived as the preferred tool over the Standard given its greater usability and ability to engage with instruction [24].

Part of the intention of the 2-phased KMb campaign was to ensure the activities of Phase 2 complemented those of Phase 1, further disseminating and promoting the tools and resources to the target audiences. This follows the KTA framework of engaging with the target demographics or audiences in various and consistent ways, as well as existing KMb literature around best practices for engaging multiple audiences, which emphasizes the importance of using multiple channels of dissemination to lay and policy audiences [43-46,49]. While Phase 1 was evidently more successful around engagement and reach than the webinar series of Phase 2, the webinars may potentially have contributed to an increase in traffic to the CCFWP course web page, with 341 sessions observed throughout the month of April 2025 (the month of Webinar 1) and 546 sessions through the month of June 2025 (the month of Webinar 3), as well as a slight increase in traffic to the GHW website, which houses many free downloadable tools. Much of the KMb work within the care economy has been geared toward engaging the community and end users (ie, CEs and their care recipients) [50-54], rather than decision-makers and stakeholders. The findings of this study provide insight into potential best practices for engaging HR, OHS, and employer audiences in CIWPs.

### Limitations

A major limitation of this paper is specific to the nature of the data collected for analysis of the KMb campaign. There was no data collected as to whether increased traffic to the CCFWP course web page, the GHW website, and the Standard and Handbook were a direct result of the campaign activities. While it is possible and likely that the KMb campaign activities increased engagement with the tools and resources of concern, given the results presented above, we cannot conclude this definitively. Further research in this area would benefit from additional data collection that can directly link KMb campaign activities to increased engagement with the selected knowledge. It is challenging to realize this for publicly available tools and resources that do not require the visitor to enter in any information. Another limitation of this paper is missing data around the engagement metrics for Article 1. This article was excluded from the organic social media posts through MCE campaigning for Phase 1, as well as the missing polling report data from Webinar 1 that is based on Article 1. The webinar polls for Webinar 1 were launched at the very end of the webinar when many attendees began to leave the webinar, resulting in zero responses to the polls as part of Webinar 1. This further informed the polls for Webinars 2 and 3, where polls were launched prior to the question-and-answer period toward the end of the webinars. Regarding the absence of organic socials for Article 1, project partner MCE was unable to collect this data for Article 1, although it was able to be collected for Articles 2‐4. This presents implications for the results of this study, as inclusion of the missing data may have provided more accurate insight into the most successful method of knowledge dissemination as part of Phases 1 and 2.

Another limitation of this study surrounds the campaign timelines; given the first 2 articles of Phase 1 were between the months of December 2024 and January 2025, they were both likely impacted by holiday periods and the start of the new year. Recognizing many working professionals are less engaged and on holiday throughout these calendar year periods, future knowledge mobilization campaigns may look toward more active periods of professional engagement. Webinar 2 had less success in recruitment than Webinar 1, which was geared toward the HR audience, as the recruitment strategy for Webinar 2 was limited to those active in or graduates of the HR programs offered through MCE, with the absence of a channel for accessing the OHS audience. Given the students from the HR programs offered through MCE likely have limited focus on or expertise in the areas of psychological OHS, this may have hindered effective recruitment for Webinar 2. As well, Webinar 3 recruitment for the small- to medium-sized business audience was focused on all business administration programs offered through MCE, which may not effectively speak to the small- to medium-sized business audience. Finally, given the limitations of access to analytics through the GHW program website (powered by MacSites), only the previous 6 months of website traffic were able to be analyzed as part of this study (February-July), rather than the entire campaign time period (December-July). Finally, while the KMb campaign was directly informed by previous work in this area [24] with regard to key stakeholder audiences to engage, the audiences were not directly involved in the development of the campaign. Future work in this area should engage stakeholders of the target audiences directly for more curated campaigning.

### Conclusion

The 2-phased KMb campaign ran from December 10, 2024, to June 30, 2025. Phase 1 of the campaign, surrounding the 4 published magazine articles, resulted in significantly higher engagement with the KMb campaign materials than Phase 2 of the campaign, which entailed a webinar series built around the articles published in Phase 1. Access to the audiences and networks of the leading national magazines for HR, OHS, and small- to medium-sized businesses in Canada potentially contributed to the observed increase in traffic to the CCFWP course web page, increased registrations to the course and student enrollments, as well as increased traffic to the GHW website, specifically around the publication date of Article 3. Across all 4 articles, Article 3 had the greatest reach to the target audience, coinciding with an increase in visitors to the CCFWP course web page and registration and enrollment throughout the entire campaign. While the Phase 1 articles performed well for the HR and small- to medium-sized business audience, Phase 2 was comparatively less successful with respect to engagement with the campaign tools amongst the target audiences. Formally publishing articles in nationally leading industry magazines that target audiences directly was observed to be the most effective method for this type of KMb focused on increasing awareness, engagement, and uptake (ie, CCFWP course enrollments and registrations and Standard and Handbook downloads). Overall, the campaign potentially contributed to increases in engagement with the tools and resources that were part of this campaign, which were designed to help organizations create more carer-inclusive workplaces across Canada. Nonacademic, industry magazine publications are an effective approach to engaging stakeholder audiences in CIWPs.

At the time of this writing, the landscape of literature surrounding KMb strategies and best practices for KMb research pitched toward stakeholders within the carer-inclusive policy space is limited. This research adds to the growing literature of KMb science with regard to best practices for engaging stakeholders and decision-makers within the carer-inclusive policy space. Specifically, more work is needed to establish causal and correlative associations, as well as engage more directly with workplaces and employers to realize a national shift toward more equitable and sustainable work for the growing number of CEs in Canada. With a rapidly aging population and increased strain on Canadian health care systems, building awareness and uptake of carer-inclusive workplace tools is imperative to support the growing number of CEs. Equipping workplaces in Canada with the CIWPs needed to sustain CE productivity and well-being through KMb science strategies presents a viable approach to mitigating these growing challenges. Further research in this area is needed to continue refining the KMb strategies that are most effective for raising awareness of CIWPs.

## Abbreviations

AARP: American Association of Retired Persons
CCFWP: Creating Caregiver-Friendly Workplaces
CE: carer-employee
CIWP: carer-inclusive workplace practices
CPR: cost per result
CRM: Customer Relationship Management
CSA: Canadian Standards Association
GHW: Gender, Health and Work
GSS: General Social Survey
HR: human resources
KMb: knowledge mobilization
KTA: Knowledge to Action
MCE: McMaster Continuing Education
MERB: McMaster Ethics Research Board
OHS: Occupational Health and Safety
ROI: return on investment
SSHRC: Social Sciences and Humanities Research Council of Canada
